# Augmented Reality Microscopy in the Management of Cerebellopontine Lesions and Microvascular Decompression: A Pilot Study

**DOI:** 10.1097/ONO.0000000000000004

**Published:** 2021-11-22

**Authors:** Lawrence Kashat, Purven Parikh, Khalil Rahman, Tessa Ryan, Denis Lafreniere, Ketan R. Bulsara, Daniel S. Roberts

**Affiliations:** Division of Otolaryngology and Neurosurgery, Department of Surgery, School of Medicine, University of Connecticut, Farmington, Connecticut.

**Keywords:** Angiography, Augmented reality, CPA neoplasm, Lateral skull base, Microscopy, Microvascular decompression

## Abstract

**Objective:**

To evaluate whether augmented reality microscopy surgical fluorescence technology, already Food and Drug Administration approved for vascular neurosurgery, can aid in lateral skull base surgery during cerebellopontine (CPA) tumor resection and microvascular decompression.

**Study Design:**

Pilot prospective uncontrolled observational cohort study.

**Setting:**

An academic tertiary care hospital.

**Patients:**

Those who underwent retrosigmoid craniotomy for CPA tumor resection or microvascular decompression for hemifacial spasm, trigeminal neuralgia or pulsatile tinnitus. 11 patients were recruited: 4 underwent CPA tumor resection and 7 underwent microvascular decompression.

**Interventions:**

Augmented reality microscopy with fluorescence imaging was utilized to visualize vascular flow intraoperatively. A postoperative surgeon questionnaire was administered to assess the intraoperative efficacy of this technology.

**Main Outcome Measures:**

Efficacy of technology in aiding with CPA tumor resection and microvascular decompression.

**Results:**

For all 7 microvascular decompression cases, surgeons agreed that the technology aided in identifying areas where disease was affecting tissues with no cases of vascular occlusion identified. In 3 of the 4 CPA tumor resection cases, surgeons agreed that the technology identified areas of vascular flow within the CPA and the tumor. Vascular patency of the sigmoid-transverse sinus was also confirmed. No significant adverse effects were noted except 1 instance of severe-to-profound sensorineural hearing loss.

**Conclusions:**

Our study shows that the augmented reality fluorescence technology works during lateral skull base surgery as it can confirm intraoperative vascular integrity. Our data also suggest that this technology may improve visualization of ambiguous vasculature and blood flow to diseased tissue.

Vascular compression of cranial nerves as occurs with trigeminal neuralgia or hemifacial spasm may cause intermittent twitching of the muscles innervated by the affected nerve and may also cause chronic pain.(1,2) In the case of hemifacial spasm, botulinum toxin can be used as medical management to improve symptoms, but relief is often temporary.(3) Similarly, prophylactic medication with sodium channel blockers can be used as initial treatment for trigeminal neuralgia.(4) Surgical intervention may be considered for those with severe symptoms or in patients who fail medical management.

Microvascular decompression through retrosigmoid craniotomy is a surgical intervention used to permanently alleviate compression of the trigeminal nerve or facial nerve by an adjacent blood vessel.(5,6) This has been shown to have the potential to provide consistent symptomatic relief.(5,6) Arterial compression occurs most frequently with the anterior inferior cerebellar artery and posterior inferior cerebellar arteries.(7) Venous compression is also possible and is likely the cause for many complications and incomplete decompression.(5,6)

Retrosigmoid craniotomy is also a commonly utilized approach to resection of cerebellopontine (CPA) tumors and requires an integrated understanding of neurovascular anatomy. In the setting of CPA tumor resection, these approaches require surgeons to rely solely on gross visualization of tumor and neural monitoring, in some cases limiting their ability to achieve total resection and increase risk of complications and tumor recurrence. One area of concern arises from an inability to determine if manipulation of vascular structures is disrupting circulation intraoperatively. In this scenario, life-threatening cerebellar and brainstem infarction is possible, with estimated risk at 0% to 2.1% for microvascular decompression.(8) Thus, an intraoperative tool to help identify vascular structures and vascular flow would help surgeons better identify and excise tumor margins and would help reduce the risk of thromboembolism and stroke after vascular manipulation.

The use of augmented reality technology to improve the intraoperative visualization of anatomical structures has been studied previously.(9) The combination of immunofluorescence imaging with augmented reality systems has been shown to improve identification of vascular structures, assessment of tumor perfusion, and assessment of tumor resection margins in laparoscopic hepatectomy for hepatocellular carcinoma.(10) A previously published case report from our institution also shows the utility of augmented reality microscopy with fluorescence imaging in the management of hemifacial spasm.(11) Our current study, however, reports on a newer type of augmented reality microscopy with fluorescence that has not yet been formally studied for use in microvascular decompression. This pilot study aims to assess the potential of this technology in enhancing the visualization of vascular structures, improving tumor resection margins, and reducing surgical complications.

## MATERIALS AND METHODS

This pilot study was a single-institution uncontrolled prospective cohort study to evaluate the potential helpfulness of the ARveo Augmented Reality Microscope with GLOW800 Fluorescence Imaging microscope accessory (Leica Microsystems, Wetzlar, Germany) during microvascular decompression and CPA angle tumor resection. Patients who underwent microvascular decompression either had trigeminal neuralgia, hemifacial spasms or pulsatile tinnitus due to vascular compression.

Intraoperatively, 12.5 to 25 cc of indocyanine green (ICG) immunofluorescence was injected intravenously immediately before the need for visualization of vascular flow. This typically occurred immediately before and after microvascular decompression took place, during and after tumor resection for CPA tumors, and when there was ambiguity regarding the presence and/or patency of vascular structures. Fluorescing vascular structures were then visualized in-vivo and in real-time within the surgical field using the augmented reality fluorescence imaging microscope accessory with the augmented reality microscope. The ICG dose was also recorded.

A surgeon questionnaire was then completed postoperatively by the operating surgeons. The neurosurgeon (K.R.B.) and neurotologic surgeon (D.S.R.) researchers assigned a consensus rating to the survey questions. The following 7 questions were answered:

1. Aid in identifying areas where disease is affecting the tissue?
2. Aid in identifying the margins of the tissue that should be removed?
3. Aid in identification of location of angiogenesis?
4. Identify other vascular flow?
5. Aid in avoiding any complications?
6. Change management?
7. Aid in more complete resection?

To create consistency, numerical values were assigned to the responses: 1 = strongly disagree, 2 = disagree, 3 = neutral, 4 = agree, 5 = strongly agree, N/A. Surgical outcomes were tracked over time, including completeness of treatment response, completeness of resection, completeness of response at most recent postoperative follow-up, complications (cerebrospinal fluid leak, facial nerve injury, hearing loss, stroke), and disease recurrence. This study was approved by the University of Connecticut Health Center Institutional Review Board in Farmington, CT; the principal investigator was Dr Denis Lafreniere, MD, and the IRB number was 20-001-1.

## RESULTS

**FIG 1.**
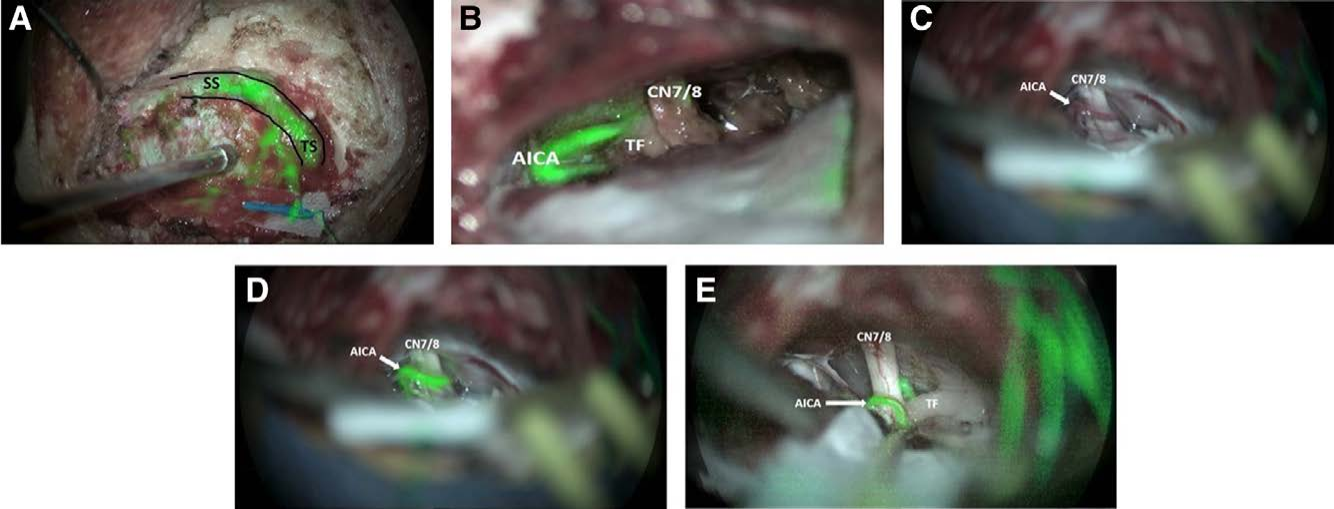
Representative photographs of augmented reality microscopy during lateral skull base surgery. *A*, Left TS and SS visualized using augmented reality microscopy. Following intravenous injection of ICG, the vessel fluoresced and was visualized with surrounding anatomy. *B*, Visualization of AICA and TF implant adjacent to the vestibulocochlear-facial nerve complex (CN 7/8) using augmented reality microscopy following decompression. *C–E*, Visualization of facial nerve and AICA loop adjacent to CN 7/8 before (*C*) and after ICG injection (*D*), and after decompression (*E*) using augmented reality microscopy. Additional compression also noted inferior to dorsal root entry zone. AICA indicates anterior inferior cerebellar artery; ICG, indocyanine green; SS, sigmoid sinus; TF, teflon; TS, transverse sinus.

This study ran from December 2018 to May 2020. A total of 11 patients were recruited for the study. Baseline demographic information is shown in Table 1. Figure 1 demonstrates intraoperative photographs which help to illustrate the surgical field. Operative findings demonstrate the ability to visualize venous blood flow, specifically the transverse-sigmoid sinus junction (Fig. 1A). In all microvascular decompression cases, the augmented reality microscope with fluorescence imaging aided in the visualization of the complex association between vascular structures, adjacent nerve complexes, and visualization of vascular flow (Fig. 1B). Notably, in addition to aiding in direct visualization of the vessel in question (Fig. 1C, D), we found that the technology can be used after a microvascular decompression to confirm vessel patency (Fig. 1E).

**TABLE 1.** Patient demographics

Total patients	11
Mean age (SD), range	59.16 (9.2), 46–73
Gender	
Male	5
Female	6
Median time for follow-up, range, mo	3, 1–17
Ethnicity	
White	8
Asian	1
Black	1
Other	1
Diagnosis	
Hemifacial spasm	4
CPA neoplasms	4
Trigeminal neuralgia	2
Pulsatile tinnitus	1
Surgery	
Microvascular decompression	7
CPA tumor resection	4

CPA indicates cerebellopontine.

Of the patients who underwent microvascular decompression for hemifacial spasm (n = 4), 1 patient was noted to have a partial response and 3 of 4 patients were noted to have a complete response (Table 2). At most recent follow-up, all patients were stable in their response with no recurrence of disease. For patients who underwent microvascular decompression for trigeminal neuralgia (n = 2), all achieved a complete response with no disease recurrence at most recent follow-up (Table 2). The lone patient with pulsatile tinnitus achieved a partial response to microvascular decompression with no worsening at most recent follow-up.

**TABLE 2.** Microvascular decompression outcomes

Patient	Involved nerve	Involved artery	Dose of ICG	Outcome	Durability of response	Follow-up, mo
B	Trigeminal nerve	AICA	25 cc	Cured	Stable	9
C	Vestibular and cochlear nerve	AICA	25 cc	Partial improvement	Stable	3
D	Facial nerve	AICA	25 cc	Partial improvement	Stable	17
F	Trigeminal nerve	AICA	25 cc	Cured	Stable	3
2	Facial	AICA	25 cc	Cured	Stable	3
3	Facial	AICA	25 cc	Cured	Stable	3
4	Facial	AICA	25 cc	Cured	Stable	2

AICA indicates anterior inferior cerebellar artery; ICG, indocyanine green.

Of the CPA tumor resections (n = 4), 2 were meningiomas, 1 was a vestibular schwannoma, and 1 was noted to be hemangioblastoma (Table 3). Complete resection was achieved in 2 of 4 patients including the 1 hemangioblastoma case and in 1 meningioma case. Subtotal resection was achieved in the remaining 2 cases.

**TABLE 3.** CPA tumor resection outcomes

Patient	Tumor type	Dose of ICG	Outcome	Evidence of disease progression at most recent follow-up?	Follow-up, mo
A	Meningioma	25 cc	Gross total	Stable	8
E	Meningioma	12.5 cc	Subtotal	Stable	9
1	Acoustic neuroma	25 cc	Subtotal	Stable	5
5	Hemangioblastoma	25 cc	Gross total	Stable	1

CPA indicates cerebellopontine; ICG, indocyanine green.

For all cases, there were no incidents of facial nerve injury, cerebrospinal fluid leak, stroke, death, or other serious adverse events. There was 1 occurrence of severe-to-profound sensorineural hearing loss which was persistent at 3 months of follow-up for microvascular decompression for hemifacial spasm.

A summary of surgeon responses to clinical questionnaire is shown in Tables 4 and 5. For all 7 microvascular decompression cases, surgeons agreed that the augmented reality microscope with fluorescence imaging aided in identifying areas where disease is affecting the tissues (Table 4). For 3 of 7 patients, surgeons agreed the technology helped identify other areas of vascular flow. For the remaining 4 of 7 cases, surgeons disagreed that it helped to identify other areas of vascular flow. Surgeons disagreed that the technology helped to avoid complications or changed surgical management in all cases.

**TABLE 4.** Microvascular decompression surgeon satisfaction survey results

Survey question	Likert scale					
	1 = strongly disagree	2 = disagree	3 = neutral	4 = agree	5 = strongly agree	Not applicable
Aid in identifying areas where disease is affecting the tissue?	-	-	-	7 (100%)	-	-
Aid in identifying the margins of the tissue that should be removed?	-	-	-	-	-	7 (100%)
Aid in identification of location of angiogenesis?	-	1 (14.3%)	-	-	-	6 (85.7%)
Identify other vascular flow?	-	4 (57.1%)	-	3 (42.9%)	-	-
Aid in avoiding any complications?	1 (14.3%)	6 (85.7%)	-	-	-	-
Change management?	-	7 (100%)	-	-	-	-
Aid in more complete resection?	-	-	-	-	-	7 (100%)

**TABLE 5.** CPA tumor resection surgeon satisfaction survey results

Survey question	Likert scale					
	1 = strongly disagree	2 = disagree	3 = neutral	4 = agree	5 = strongly agree	Not applicable
Aid in identifying areas where disease is affecting the tissue?	-	4 (100%)	-	-	-	-
Aid in identifying the margins of the tissue that should be removed?	-	4 (100%)	-	-	-	-
Aid in identification of location of angiogenesis?	-	4 (100%)	-	-	-	-
Identify other vascular flow?	-	1 (25%)	-	3 (75%)	-	-
Aid in avoiding any complications?	-	4 (100%)	-	-	-	-
Change management?	-	4 (100%)	-	-	-	-
Aid in more complete resection?	-	4 (100%)	-	-	-	-

CPA indicates cerebellopontine.

For 3 of the 4 patients with CPA tumor resections, surgeons agreed that the technology identified other areas of vascular flow (Table 5). Surgeons disagreed that the technology aided in identifying affected tissue, margins of resection, location of neoangiogenesis, avoidance of complications, completeness of resection, or resulted in change to surgical management.

## DISCUSSION

ICG is increasingly being used in neurovascular surgery for fluorescence-guided tumor resection, but there are few publications about the effectiveness of this procedure.(12) ICG angiography has been reported to safely resect tumors by identifying healthy tissue and outlining the margins of the tumor.(12) Our study introduces a novel use of ICG fluorescence technology by describing the first series of use of augmented reality microscopy using a novel fluorescence technology. This study differs from all previous reports using ICG as this is the first report to describe the use of a microscope accessory which allows for the real-time visualization of fluorescing vascular structures. The technology allows for immediate viewing of vascular flow which is superimposed on the natural microscope view of the surgical bed (ie, there is no need to switch to an infrared camera to view vascular flow and subsequently switch back to the traditional white light view with the microscope).

Our study found that augmented reality microscopy with fluorescence imaging technology aided in tumor resection in some cases, a finding similar to that observed previously.(12) Our surgeon response data for CPA tumor resection also suggests a possible role for this technology in helping identify areas of increased vascular flow. This is similar to another study involving hemangioblastoma resection in which authors reported that ICG angiography provides useful information on hidden vessels and transit feeders to aid in tumor resection.(13) In our data set, surgeons provided higher ratings for vascular identification in the sole hemangioblastoma case, but surgeons disagreed that it helped identify blood flow that was not already apparent to the surgeon for the remaining cases. Although our study does not report tumor volumes, an additional application of this technology could be in the identification of blood vessels that are running through the tumor beds of larger CPA tumors with more challenging anatomy. Further research will need to be completed to assess whether this technology may help improve outcomes in this setting. Future research should also investigate whether this augmented reality technology can help decrease operative times and cognitive load by obviating the need to switch imaging modalities and to reorient anatomic structures.

As explored in our study, augmented reality angiography with ICG immunofluorescence can also assist in microvascular decompression to identify the offending vessel and its location relative to the affected nerve. Von Eckardstein et al.(14) reported that ICG angiography can be helpful in decompressing the trigeminal nerve and identifying the nerve-vessel conflict in microvascular decompression for trigeminal neuralgia. This study differed from our current study in that the authors had utilized angiography which required the use of near-infrared light. This requires switching between white light visualization to a near-infrared detector which often decreases the visualization of other nonvascular structures within the surgical field. In contrast, our microscope accessory allows for the simultaneous visualization of vascular flow which fluoresces after ICG injection and is visualized as an augmented reality image within the surrounding tissue (Fig. 1). Both our study and the report by von Eckardstein et al.(14) used ICG before and after decompression and demonstrated that ICG angiography is useful in microvascular decompression (MVD) to confirm vascular flow.

An additional application of ICG in microvascular decompression is its use during dural opening, which our study examined in 1 case (Fig. 1). Yokoyama et al.(15) reported that ICG videoangiography can be used before dural opening in MVD to identify the margins of the dural sinus, thus increasing the safety of the dural opening. Our study demonstrates a way of obtaining the benefits of angiography while not requiring a switch to near-infrared light due to the fluorescent 3-dimensional augmented reality image that is obtained.

Using this augmented reality imaging is effective in identifying vascular flow, but it is also valuable in that it can aid in avoiding injury of both the sigmoid sinus and the transverse sinus. Ohata et al.(16) looked specifically at occlusion of the sigmoid sinus, and the symptomatic consequences following surgery. In various dominant sigmoid sinus occlusions, patients experienced severe intracerebral hemorrhages, supra- and infratentorial brain edema, and hemorrhagic infarction with aphasia; these were caused by either sinus laceration or sinus compression.(16) ICG has the potential to illuminate the sigmoid sinus and transverse sinus, which could further prevent complications to these vessels during surgery. Our surgeon questionnaire data also suggest that ICG used in conjunction with our microscope accessory has the potential to aid in identification of other areas of vascular flow and areas where disease is affecting tissue, particularly in microvascular decompression cases. As visualized in Figure 1C–E, this technology allows for the immediate confirmation of vessel patency.

Our pilot study found that ICG was helpful in identifying where disease was affecting tissue in microvascular decompression, but not in the case of CPA tumors. It is possible that these findings may change with more highly vascularized tumors, tumors that are larger in size, or with different tumor histopathology. Importantly, these conclusions are based on a preliminary analysis of a small sample of patients, so further research with a larger patient cohort is needed to further assess these findings.

Augmented reality imaging has potential uses that extend beyond those described in this study. A common complication of CPA surgery is postoperative hearing loss caused by damage to the labyrinthine artery.(17) The labyrinthine artery is responsible for cochlear blood flow, and restriction of this blood flow during surgery can cause hearing loss.(17) A potential application of ICG is to use it to monitor the labyrinthine artery and assess its integrity during surgery. The use of ICG could potentially detect any fluctuations labyrinthine artery blood flow, which could predict hearing loss outcomes. In our study, the technology has proven to be a reliable form of angiography. Evaluation of blood flow using this technology as performed in our study is qualitative in nature as evidenced by the flow of fluorescent dye through the blood vessel. Quantitative assessment of vascular flow to blood vessels is certainly an interesting idea for future research. For cases of pulsatile tinnitus requiring decompression/surfacing of a sigmoid sinus diverticulum, use of ICG could aid in confirmation of patency during surgery. Finally, while not the subject of our study, use of ICG may aid in decompression of the sigmoid sinus in cases of sigmoid sinus thrombosis, possibly showing real-time improvement of vascular flow.

It is important to acknowledge the limitations of our pilot study which include the small sample size, the fact this study was completed in a single institution, the lack of control group and patient randomization, and the subjective nature of the survey instrumentation. Nevertheless, our study provides some preliminary useful information on potential applications of a novel technology. We have demonstrated that the technology works during these surgeries and has potential benefit which requires further studies to evaluate. Accordingly, this pilot study is meant to describe the use of a novel technology and will require further study to determine its clinical application with microvascular decompression and CPA tumor resection. It is important to note that future research will also need to be completed to determine the sensitivity of the technology at detecting smaller caliber vessels and what this size limit may be.

## CONCLUSION

This report presents the first prospective cohort study describing the use of ICG immunofluorescence angiography in combination with augmented reality microscopy in patients undergoing microvascular decompression or CPA tumor resection. Our pilot study demonstrates the feasibility of this technology during microvascular decompression as it assists in identifying vascular structures and their location relative to affected nerves. Furthermore, because it can immediately confirm vascular patency, it may help identify areas of vascular damage intraoperatively and it appeared to aid in the identification of vascular structures and vascular flow within tumors. Overall, we believe that there is a role for this technology in improving visualization of ambiguous vasculature and blood flow to diseased tissue. This will require further evaluations and research to help determine the possible uses of this innovative technology. The data must be interpreted with caution due to the lack of control group and randomization, small nature of this pilot study, and subjective nature of survey instrumentation. Future studies will help to more clearly delineate the possible benefits and uses of this novel technology.

## FUNDING SOURCES

Funding by the 2019 New England Otolaryngological Society Resident Research Grant by the New England Otolaryngological Society. A total of $5000 was awarded to Lawrence Kashat, MD, MSc, Denis Lafreniere, MD, and Daniel S. Roberts, MD, PhD.

## CONFLICT OF INTEREST

None declared.
